# DODO: an efficient orthologous genes assignment tool based on domain architectures. Domain based ortholog detection

**DOI:** 10.1186/1471-2105-11-S7-S6

**Published:** 2010-10-15

**Authors:** Ting-wen Chen, Timothy H Wu, Wailap V Ng, Wen-chang Lin

**Affiliations:** 1 Institute of Biomedical Informatics, National Yang-Ming University, Taipei, Taiwan; 2 Institute of Biomedical Sciences, Academia Sinica, Taipei, Taiwan

## Abstract

**Background:**

Orthologs are genes derived from the same ancestor gene loci after speciation events. Orthologous proteins usually have similar sequences and perform comparable biological functions. Therefore, ortholog identification is useful in annotations of newly sequenced genomes. With rapidly increasing number of sequenced genomes, constructing or updating ortholog relationship between all genomes requires lots of effort and computation time. In addition, elucidating ortholog relationships between distantly related genomes is challenging because of the lower sequence similarity. Therefore, an efficient ortholog detection method that can deal with large number of distantly related genomes is desired.

**Results:**

An efficient ortholog detection pipeline DODO (DOmain based Detection of Orthologs) is created on the basis of domain architectures in this study. Supported by domain composition, which usually directly related with protein function, DODO could facilitate orthologs detection across distantly related genomes. DODO works in two main steps. Starting from domain information, it first assigns protein groups according to their domain architectures and further identifies orthologs within those groups with much reduced complexity. Here DODO is shown to detect orthologs between two genomes in considerably shorter period of time than traditional methods of reciprocal best hits and it is more significant when analyzed a large number of genomes. The output results of DODO are highly comparable with other known ortholog databases.

**Conclusions:**

DODO provides a new efficient pipeline for detection of orthologs in a large number of genomes. In addition, a database established with DODO is also easier to maintain and could be updated relatively effortlessly. The pipeline of DODO could be downloaded from http://140.109.42.19:16080/dodo_web/home.htm

## Background

Orthologous gene identification is an important step in comparative genomics. The word orthologs originally refer to genes in different species derived from the same locus in their last common ancestor. Since orthologs are genes derived from the same ancestor gene, orthologs often have similar amino acid sequences and expected to perform the same or similar cellular function [1,2]. These properties make orthologs useful in functional genomics analysis. In addition to reconstructing the phylogeny and revealing the evolution history of species, orthologs could also be applied to genome annotation and protein-protein interaction prediction [3,4]. The orthologs can be treated as corresponding genes in different species after species evolved and consequently it is an important issue to detect this kind of ortholog relationship between species.

A number of methods have been developed for orthologs detection[5]. In practice, orthologs are defined through reciprocal best hits (RBH) from primary protein sequences between two species by various algorithms. For instance, the COG, InParanoid, and orthDB are built based on such RBH approach [6-8]. Beside RBH, tree-based methods such as those for reconstructing the LOFT, COCO-CL and HOPS database have also been developed [9-11], where trees are established via heuristic calculations of sequence similarity and the orthologous relationships are inferred from the tree structures. Some databases such as the Ensembl Compara and HomoloGene are constructed with both RBH and phylogenetic tree information [12,13]. In addition, some methods identify orthologs by reconstructing genome rearrangement events in closely related genomes such as MSOAR and MultiMSOAR [14,15].

With the advance of high throughput sequencing technologies, it is anticipated a dramatic increase in the number of completed genomes. Two challenges are posed to ortholog identification. The first issue is the speed of analyzing a large number of proteins. Increasing number of genomes necessitate faster method for data analysis and processing. Another issue is the ability to identify orthologs in distantly related species where sequence similarity might be low. However, the complexity and computation time of the RBH methods increase considerably as mutual comparisons are needed between each pair of species. For example, it needs 4,950 times of mutual comparisons between pair of genomes to identify ortholog relationships among 100 genomes and for 1000 genomes it would need 499,500 times of sequence comparison and alignments. Thus, new methods that can identify orthologous relationships among a large number of genomes, some of which are distantly related, in a reasonable time are beneficial. Here we propose an efficient and function-based new ortholog detection method called DODO (DOmain based Detection of Orthologs) to overcome the hurdles in ortholog identification from a large number of genomes.

DODO pipeline is designed for efficient discovery of the orthologous relationship between an anchor genome of interest (or well studied) and other genome(s). DODO detects homolog groups aided by protein domain information. In the beginning, DODO classifies proteins into groups based on both their domain composition and architecture. Domains are the functional units of proteins. Proteins having the same domain architecture likely have the same cellular function which implies homology in structures and functions. While the similarity between primary sequence of orthologs may decrease dramatically in distantly related species, the domain composition is more likely to be conserved through evolution due to the functional constraint [16,17]. The domain architecture based method could be applied to detect homologous relationships between distantly related species. After proteins of the same domain architecture are grouped together, DODO further refines the orthologous relationship within each homolog group by identifying RBH among the smaller protein set. This strategy of ortholog searching in smaller groups instead of the whole genome makes DODO an efficient pipeline.

In addition to efficiency, database established by DODO could also be easily updated and practically the DODO results are comparable to those predicted by the traditional RBH methods. Adding new species into the database does not require reprocessing of he previously analyzed species which already existed in the database - a procedure necessitated by the traditional RBH methods. For traditional RBH methods, to update a database consisted of n existed old species, the newly added m species will cost n*m times of mutual comparisons between each pair of existed old genomes and newly added genomes. Instead, to update a database constructed by DODO only needs m times of domain identification for those newly input genomes no matter how many species already included in the database. It is easier to maintain and update an ortholog database efficiently in this schema.

## Implementation

The DODO pipeline, which can be freely download and executed locally, is written in Python. Given input the protein sequences in FASTA format, the pipeline will run RBS-BLAST, cluster the proteins with the same domains, and finally output a report the ortholog groups automatically. DODO requires BLAST for domain identification and similarity search. The ortholog group assignment is done in two steps. Proteins are assigned into homolog groups based on their domain information and then further classified by RBH within homolog groups.

### Grouping of proteins according to domain architecture

Domain assignment is performed with RPS-BLAST for each protein sequence using Pfam v23 [18] as the source database. Default parameters are used except the expected value which is set to below 0.01. Domain hit(s) information is then extracted from hits in the RPS-BLAST result files. Proteins having the same domain composition and order are grouped together into one group. Proteins without Pfam domain information are all grouped into an uncharacterized group for further analysis.

### Assigning the ortholog group

For some of the proteins, the information of protein domain alone may not be sufficient to determine the orthologous relationship. These groups may contain the same protein architecture, but some of them may nevertheless be very different at the sequence level and thus their ortholog relationship could be resolved. This is especially evident on expended paralogous gene families. Therefore, proteins within the same domain architectural group are further sub-classified with the RBH method. Choosing one species as anchor, BLASTP is performed to identify RBH between the anchor species and all the other species. These final sets of groups are then reported as the ortholog groups.

### DODO Output

The output of DODO pipeline is a text file containing the ortholog information. Orthologs identified based on both domain information and RBH have IDs starting with 'PfamArcNu' while orthologs identified based purely on RBH have IDs starting with 'NoDomainInfo'. The domain architecture for orthologs could be found in the file PfamArcMap.txt under the project folder.

## Results

DODO first clustered proteins into groups based on their domain architectures and then found orthologous relationship within each group. This strategy speeds up the ortholog identification procedure and facilitates the maintenance of ortholog database. Here we investigate the efficiency of DODO and compare the performance of DODO against published databases.

### Computation time comparison

A dataset of 21,673 human and 23,497 mouse protein sequences used in InParanoid [7] is utilized to demonstrate the relative short processing time of DODO. The comparison was done on a Linux server with 16GB RAM and 4*AMD Operon CPU. The total computation time of DODO was 21,263 seconds (5.91 hours) while the InParanoid pipeline took 135,585 seconds (37.66 hours). This result shows that, even considering only two species, DODO can identify the orthologous relationships within these species in about 15.7% of the time that the conventional RBH takes. This difference in computation time will become larger as more species are analyzed. The computation time of the conventional RBH method grows roughly proportionally to the square of the number of species. On the other hand, DODO compares each species to the same domain database only once, regardless of how many species were in comparison. Therefore DODO has significant advantage over conventional RBH in terms of the process time. This is increasingly important as more and more genomes are being sequenced and analyzed today than ever before.

### Comparison of DODO ortholog groups with the HomoloGene release 64

HomoloGene [13] is a homolog sequence database which was constructed based on both sequence information and phylogeny information. It records the homolog relationship between 20 completely sequenced eukaryotic genomes. We extracted the 300,701 protein sequences that are used in HomoloGene release 64 from RefSeq and those sequences are a subset of a total of 330,610 protein sequences originally used in HomoloGene release 64 reconstruction. Using human as the anchor species, DODO identified 18,202 ortholog groups. These cover 92.7% of homolog groups containing human proteins in the HomoloGene dataset. We investigated whether those ortholog groups identified with DODO was a subset of groups reported in HomoloGene. Since HomoloGene is a database of homologs, each group in HomoloGene is likely to be a superset of orthologs. We found that 46.7% of ortholog groups identified with DODO have exactly the same classification as HomoloGene and 89.5% of them have more than half of the proteins present in the corresponding ortholog groups in HomoloGene 64.

**Figure 1:**
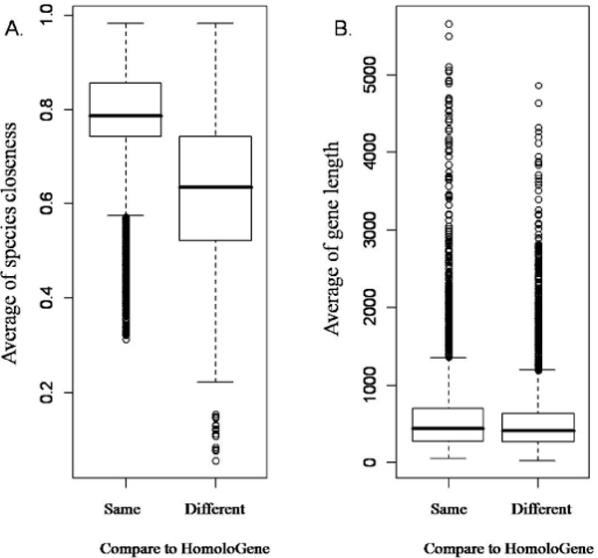
**Species closeness and gene length of the ortholog groups identified with DODO**. There are two set of ortholog groups identified with DODO, when compare to HomoloGene database. One set of them (n = 8507) has same classification as HomoloGene and the other set of them (n = 9695) has different classification from HomoloGene. (A) The closeness of each ortholog group in these two sets was calculated according to the similarity of taxonomy as described in NCBI. The set of same classification was significantly higher than the different set (wilcoxon test, p-value < 2.2e-16). This result shows that part of ortholog groups identified with DODO contains putative orthodox from distantly related species. (B) The average gene length was calculated for each ortholog group in either the same classification or different classification set. The set of same classification had significantly longer average gene length than different classification set (wilcoxon test, p-value = 8.93e- 10). This implied that DODO did find some ortholog groups composed of shorter sequences. Those shorter sequences may contain insufficient information; therefore, their orthologous relationship could not be found by conventional RBH ortholog detection method.

Since previous domain rearrangement study showed that most domain fusion events happened once in the protein evolution history [19], orthologs sharing the same domain architecture identified with DODO but not in HomoloGene database may be putative orthologs. We speculated the reason of why these putative orthologs cannot be detected solely by primary sequences is possibly due to short sequence length or low sequence similarities which may be rescued by considering domain information. Further statistical analysis indicated that those ortholog groups were composed of significantly shorter sequences and distantly related species as shown in Figure 1. Those orthologs may be rescued when considering their domain information. This fits in with DODO's assumption that domain should be more conservative than primary sequences, and taking those into consideration may increase the sensitivity in ortholog detection.

### Comparison of DODO ortholog groups with InParanoid

**Figure 2:**
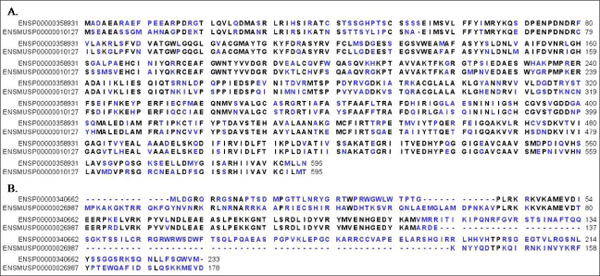
**examples of putative ortholog group found by DODO**. Two examples of ortholog groups found with DODO which are not recorded in InParanoid. The alignments were generated by CLC free Workbench version 4.0.2. Consensus residues are shown in black and dissimilar residues are shown in blue. (A) These two sequences are clustered together with DODO and both are reported to have four different domains: Transketolase_N/E1_dh/Transket_pyr/Transketolase_C. (B) These two sequences are the only protein containing the Nop16 domain in human and mouse genomes.

InParanoid [7] is a well known database established based on primary sequence comparison and including in-paralogs into ortholog clusters. Among the 21,673 human and 23,497 mouse protein sequences downloaded from the InParanoid website [7]. DODO identified 14,128 ortholog groups and 95.8% of them have the same classifications as the InParanoid. Approximately 16.6% of the orthologs recorded in InParanoid were not found in our results. Of these, most of them (98%) were composed of proteins having different domain architectures identified with RPS-BLAST. Those orthologs with apparently different domain architecture may be generated through domain rearrangement events in the protein evolution history or one or more of its domains were below the RPS-BLAST e-values cutoff. Our method is able to identify 244 ortholog groups not reported in InParanoid. Most of them are members of large protein families or proteins with short-sequences (47% of them have sequences shorter than 300 amino acids). Ortholog discovery among big family proteins can introduce complication that obscure true orthology, since true orthologs may not be reciprocally most similar in their primary sequences. One such example is shown in Figure 2A. Here, we have two putative orthologs containing the same four-domain architecture. The BLASTP procedure used in InParanoid did not find them in the RBH when searching through the entire genomes since their primary sequence similarity is relatively low when compared to some other proteins. As a result, both proteins are omitted in the In Paranoid data. However, given that they both contain the same four domains it is likely that they were functionally closely related. When domain-architecture clustering is applied prior to the RBH procedure as we did, the orthologous relationship between them could be recovered. In addition, other ortholog pairs we discovered are short sequences. The pair of ortholog sequences shown in Figure 2B is putative orthologs having difference in their protein lengths. These two sequences both contain the Nop16 domain. The Nop16 containing protein is only identified exactly once in human and mouse genomes; therefore, the two sequences are very likely to be orthologs. We checked the BLASTP results from InParanoid and found these two genes are RBH. However, InParanoid requires the matched region to be longer than 50% of the sequences in order to avoid matching at domain-level instead of finding real ortholog pair [7,20]. This might be the reason for these orthologs missed in InParanoid and we were able to discover them here.

### Orthologs detection in 100 genomes in InParanoid

**Figure 3:**
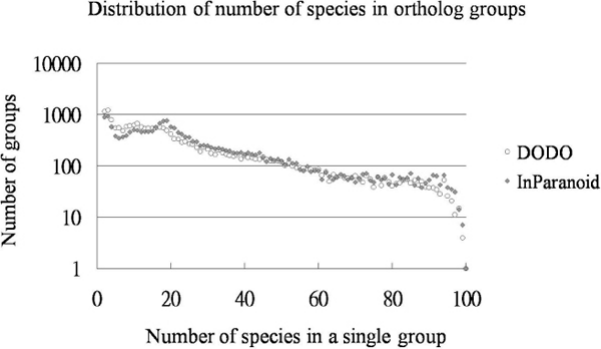
**Distribution of the number of species in ortholog groups identified with DODO and InParanoid**. Ortholog relationship between human proteins and proteins in the other 99 species (including 1 prokaryote, 17 protists, 21 fungi, 7 plants, 35 invertebrates and 18 vertebrates) were identified with DODO or downloaded from InParanoid. The horizontal axis represents the number of species in one ortholog group, and its maximum number is 100, which mean this ortholog group containing orthologous genes in all 100 species. The vertical axis represents the counts of ortholog groups in logarithmic scale. The distribution patterns are similar between DODO and InParanoid.

The species distribution of ortholog groups from DODO was compared to those from InParanoid and the distribution patterns are highly alike. Orthologous relationships between human and the other 99 genomes including fungus, plant and animal genomes are downloaded together with their protein sequences from the InParanoid website. Genes from different genomes were grouped together if they have the same ortholog gene in human genome. After this grouping step, there are a total of 20,572 ortholog groups in the InParanoid dataset. From the same dataset, DODO identified 20,461 ortholog groups by using human as anchor genome at its second RBH step. These ortholog groups contain at least 2 species and up to 100 species in a single group. The distribution of number of species in each ortholog group is show in Figure 3. The distribution of DODO and InParanoid are highly similar. There are lots of ortholog groups containing only 2 species, most of which are ortholog pairs between human and chimp. The count of ortholog groups containing 19 species is relatively abundant. This is explainable since there are a total of 19 vertebrates (including human) in the dataset. The count of ortholog groups containing more than 80 species decreases dramatically.

**Figure 4:**
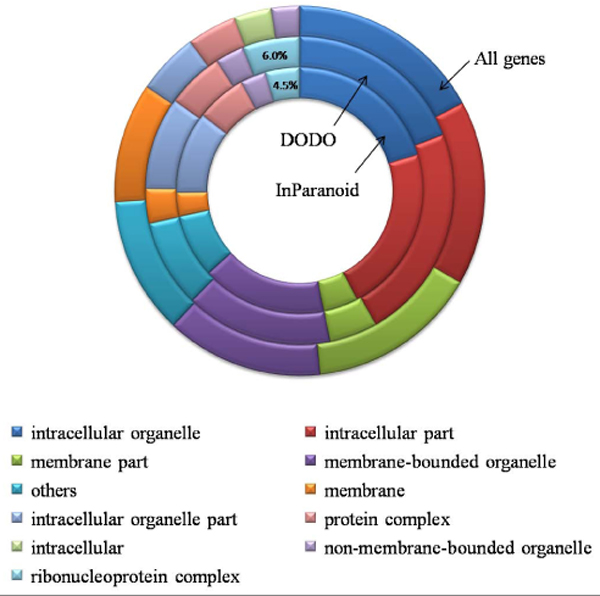
**Distribution of GO annotation (cellular component)**. Distribution of the GO annotation (cellular component) of all genes and genes having ortholog groups exist in more than 80 species in DODO output or InParanoid. Only the top 9 categories are retained and all others are merged into the category "others".

To evaluate the validity of those novel distantly-related orthologs found in this study, we assess the similarity of the Gene Ontology (GO) annotations between orthologs discovered via DODO and those found in the InParanoid database. Our discovery of orthologs should be meaningful when similarity of GO exists in contrast to the background set of human proteins. Since we were interested in the performance of DODO on ortholog detection in distantly related genomes, we focused on orthologs that were found in many species. Among the ortholog groups, there are 955 and 739 ortholog groups containing orthologs from more than 80 genomes - the "80+ ortholog groups" - in the InParanoid database and the DODO output, respectively. This means 955 (or 739) proteins in human have orthologs in more than 80 species out of the 100 species according to InParanoid (or DODO output result). These proteins are thought to participate in certain biological processes that could be very important in many different organisms; therefore, they are conserved in most of the genomes ranging from fungi to animals. Using the gene ontology (GO) of human proteins [21], we cluster the ortholog groups into different GO cellular component categories. The top 9 cellular component annotations of the 80+ ortholog groups are shown in Figure 4. The relative abundance of the 80+ orthologs groups obtained by DODO and InParanoid are similar but both are different from the background of all genes. Comparing the 80+ groups to the background set of proteins, there is enrichment for ribonucleoprotein complex, which have 6.0% and 4.5% in DODO and InParanoid, respectively. Meanwhile, there is less 80+ groups participate in membrane and intracellular categories comparing to the background.

Furthermore, due to the conservation of functions among orthologs, we expect that the domain-based strategy can expand the set of orthologs found in distantly related genomes. Upon the comparison of the human genes in the 955 and 739 80+ ortholog groups identified from InParanoid and DODO, we found 446 overlapping genes and some extra ortholog relationships which were only detected by DODO. For those 239 extra ortholog groups, about one fourth (24.9%) have the same domain architecture as those found in the 446 overlapped genes. Some (13.7%) of those have comparable 80+ ortholog groups in InParanoid but do not contain the same human genes. Lots of them (41.1%) have comparable ortholog groups from 70 to 80 species in InParanoid. Those ortholog groups could be viewed as expansion of existing ortholog groups in InParanoid after the incorporation of domain information. DODO did report some short sequence orthologs which have comparable ortholog groups covering less than 60 species in InParanoid (Table 1).

**Table 1:** Examples of DODO identified ortholog groups that were not identified in InParanoid.

Ensembl human gene id	number of species	Average a.a. length	domain	
ENSP00000375160	96	199.7	Ribosomal_L22	
ENSP00000348580	94	118.8	Ribosomal_S26e	
ENSP00000307786	91	110.7	Cytochrom_C	
ENSP00000236900	91	122.6	Ribosomal_S25	
ENSP00000337019	90	99.9	Ribosomal_S21e	
ENSP00000316649	89	477.6	Oxysterol_BP	
ENSP00000158771	87	242.2	DER1	
ENSP00000280665	87	413.9	DCP1	
ENSP00000360803	86	164.5	zf-DNL	
ENSP00000352137	86	298.6	Fcf2	
ENSP00000359368	83	461.7	RPAP2_Rtr1	
ENSP00000254101	81	314.4	AMPKBI	
ENSP00000253719	81	399.1	Asp	
ENSP00000380214	80	507.4	Sugar_tr	

Examples of the 80+ ortholog groups found by DODO but not in InParanoid. Their average amino acid length and domain composition are shown.

## Discussion

DODO detects ortholog based on domain compositions instead of primary protein sequences and has brought up several advantages in the aspect of biology. As shown in the results above, DODO was able to detect most orthologs in several published databases. In addition, it can detect orthologs having short sequences and lower sequence similarity if information of the domain architecture is evident. This strategy finds orthologs based more directly on functional constraints. As a result, ortholog groups detected with DODO are thought to have similar if not the same biological functions in organisms. Ortholog detected by this strategy will be helpful in the annotations of newly sequenced genomes of which the functions of genes are interested. The domain compositions of proteins should be more conserved than primary sequence since the sequence of proteins are susceptible to mutation while the function of proteins are under greater constraints. The protein domain composition is responsible for protein function and is thus more likely to be conserved than primary sequences in distantly related genomes.

In addition to the relative high efficiency of DODO, an orthologous database built with DODO is less costly to maintain comparing to other methods. When a new genome is added to the database, sequences of this genome could be assigned into their homolog groups based purely on their domain architecture without searching through existing genomes. Further ortholog assignment could be simply achieved through the sequence comparison between the sequence(s) from the newly input genome and the sequence from anchor genome within each homolog groups. The two-step approach of DODO will largely reduce the computation complexity when an established database is updated.

**Figure 5:**
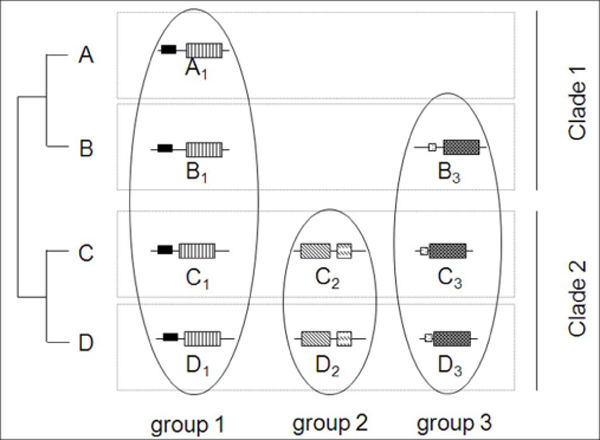
**Choosing more than one anchor genomes can rescue missing ortholog groups**. This cartoon figure illustrated examples of three different ortholog group distributions in four species A, B, C and D. Four rectangles in gray line stand for four different genomes. Protein sequences and domain are shown as line and rectangles. As shown in the figure, there are total three different ortholog groups in which group 1 exist in all genomes, group 2 is a clade 2 specific ortholog group and group 3 had a gene miss event in genome A. When choose species A in clade 1 as the anchor genome, DODO will only report group 1 and both group 2 and group 3 will be missed. Those missing ortholog groups could be identified if choose multiple genomes as anchor genomes in DODO pipeline.

The results also show that DODO is useful in ortholog detection between distantly related genomes. For a database having multiple genomes, specifically multiple distantly related genomes, it is conceivable that detection of ortholog groups may not be sufficient by a single anchor genome. There are some clade-specific genes which essentially do not have ortholog relationship to genomes in other clades. A clade-specific ortholog group can only be detected when choosing a genome within that clade as an anchor genome. For those genes, the ortholog relationship can be rescued by setting more than one anchor genome. As an example shown in Figure 5, the clade 2 specific ortholog group - group 2, could be rescued if choose genome in clade 2 (genome C or genome D) as extra anchor genome. As show in Figure 5, this strategy could also be useful in the event of gene loss in the anchor genome.

A few limitations do exist with our method. Since DODO detects ortholog based on the domain architecture, the accuracy and sensitivity of domain identification directly affect the performance of DODO. DODO cannot detect orthologs having different reported domain architecture. Indeed, these phenomena can explain most ortholog groups reported by InParanoid but cannot be found with DODO as shown in the results. There are also sequences having domain(s) on only a small part of the sequence, which may lead to a wrong homolog group classification and end in no orthologous relationship identified. This limitation of protein domain information is inherent in the method thus cannot be avoided. However, this limitation will be improved as new domains are identified, less characterized domains, such as PfamB are used or domain detection method is improved in the future. As we can expect, removing the redundancy in domain database or considering the domain match length may improve the domain identification on proteins [22].

In summary, DODO could efficiently detect orthologs having the same domain architecture even when these orthologs have short sequences or low sequence similarity. Those same domain architecture orthologs are likely to perform the same biological function and could be beneficial in annotation of newly sequenced genome. An ortholog database built by DODO is easy to update. However, the performance of DODO is highly dependent on the domain detection step.

Several protein evolution events increase the difficulty of ortholog detection, such as gene loss, gene duplication and domain rearrangement [5]. Gene loss events are known to hinder detection of ortholog in many RBH based methods. For DODO, if it occurs in genomes other than the anchor genome, this will not have significant influence on the prediction results. However, if gene loss occurs in the anchor genome, DODO could not detect ortholog relationships since there is no corresponding gene to start with in the anchor genome. This kind of missing ortholog group can be completely avoided by taking multiple genomes as the anchor genomes as shown in Figure 5. Even though there was a gene lost event in genome A, the ortholog group 3 could be identified while take other genome as the anchor genome. In the case of gene duplication, there are two different kinds of duplication. One is in-paralog, where duplication happened after the separation from the common ancestor and the other is out-paralog, where duplication happened before the speciation. For out-paralogs, DODO can detect them as separate different ortholog groups only if there was no gene loss or domain changing event. However, in the in-paralog DODO can lose one (or several) of the in-paralog(s), since DODO only keeps the RBH in the final report. That is, only the most similar in-paralog will be included in the ortholog group. Still the in-paralogs will be classified into the same domain architecture group. For the domain rearrangement events, there are tree-based methods RIO and Orthostrapper which already have been used to build ortholog relationships at the domain level [23,24]. These two methods generate confidence values from ortholog bootstrap support. Orthostrapper is used to build the HOPS database[10], which is a orthologous protein domain database. RIO and HOPS built ortholog relationships at the domain level instead of the protein level and need taxonomic information in advance while DODO built ortholog relationship between proteins and does not require the taxonomy information. Indeed, our ortholog detection is heavily based on domain architecture; hence it is affected by evolutionary events such as domain rearrangement, domain deletion or domain insertion event. DODO cannot detect orthologous relationships if there are those domain changing events in the evolution histories of the proteins.

## Conclusions

An efficient and sensitive ortholog detection method DODO is proposed. DODO could be useful in ortholog relationship construction or update of ortholog relationships especially when taking lots of organisms into consideration. In addition, most orthologous relationships detected with DODO are composed of the proteins having the same domain composition. Ortholog detection based on domain information may disclose the more biologically meaningful ortholog groups. This ortholog identification tool will be useful for those newly sequenced genome annotations using well studied genome as anchor. Indeed, DODO was able to detect most ortholog groups recorded in the known orthologous databases as well as discover new ortholog groups having relative short or dissimilar sequences but the same domain architecture. Given the high efficiency and sensitivity, DODO could be a useful method to analyze sequences produced from many genome projects.

## Availability and requirements

Project name: DODO

Project home page: http://140.109.42.19:16080/dodo_web/home.htm.

Operating system: Linux, Mac OS X

Programming language: Python

Software requirements: installation of BLAST

Restriction: none

## Authors' contributions

TC implemented, tested DODO and wrote the manuscript. TW run the GO analysis. WN and WL supervised and revised the manuscript. All authors had read and approved the manuscript.

## Competing interests

The authors declare that they have no competing interests.
